# Advances in cancer therapy: clinical benefit of new cancer drugs

**DOI:** 10.18632/aging.204839

**Published:** 2023-06-19

**Authors:** Daniel Tobias Michaeli, Julia Caroline Michaeli, Thomas Michaeli

**Affiliations:** 1 Department of Personalized Oncology, University Hospital Mannheim, Heidelberg University, Mannheim, Germany; 2 Schumpeter School of Business and Economics, University of Wuppertal, Wuppertal, Germany; 3 Department of Obstetrics and Gynaecology, LMU University Hospital, LMU Munich, Germany; 4 DKFZ-Hector Cancer Institute at the University Medical Center Mannheim, Mannheim, Germany; 5 Division of Personalized Medical Oncology, German Cancer Research Center (DKFZ), Heidelberg, Germany

**Keywords:** cancer drug, clinical benefit, overall survival, progression-free survival, clinical trial

Cancer is among the leading causes of death worldwide, with the Global Burden of Disease study estimating a total of 10 million people die of cancer yearly. Age is a major risk factor for cancer given that most cases occur in patients over the age of 65. As the global population ages, the burden of cancer on society increases. The World Health Organization forecasts that there will be 19.3 million new cases and 9.6 million deaths by 2025.

However, recent advances in medicine have led to declining mortality rates. The American Cancer Society’s recent report of Cancer Statistics 2023 estimates that overall cancer mortality has declined by 33% since 1991 [1]. Besides improvements in prevention and early detection of cancer, novel anti-cancer therapies have significantly contributed to the observed declining death rates. These novel treatments have particularly improved survival rates among patients with leukemia, melanoma, kidney cancer, and lung cancer.

**Figure 1:**
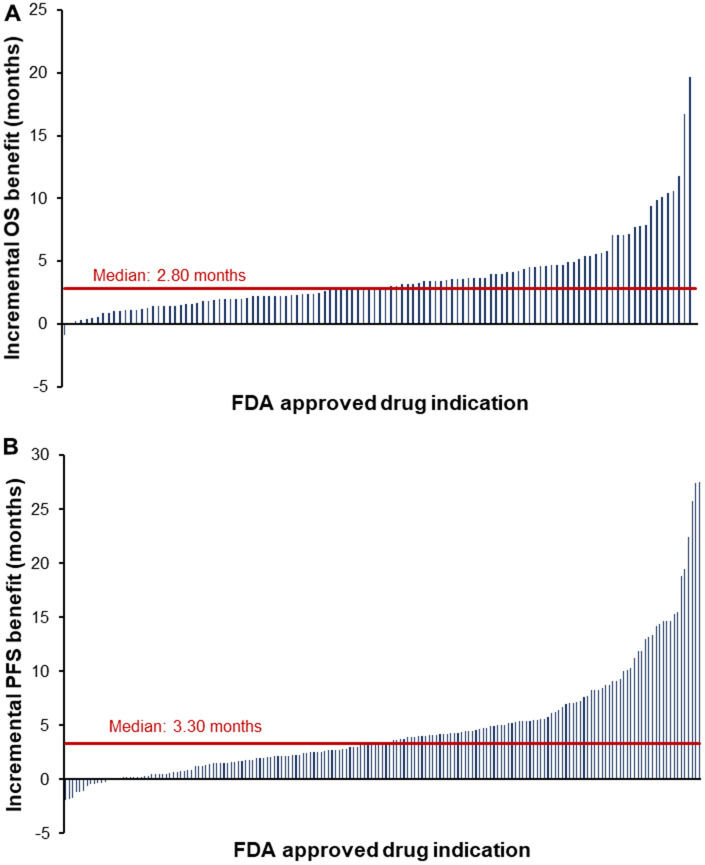
**Clinical benefit of new cancer drug indications approved by the FDA from 2003 to 2021.** Notes: The waterfall plots visualize the clinical benefit of new cancer drug indications approved by the FDA between 2003 and 2021. Each blue bar represents the benefit of a single drug indication that was measured in the pivotal randomized controlled trial supporting the FDA approval. The red line indicates the median survival benefit. Graph (**A**) show the OS benefit and graph (**B**) the PFS benefit. In graph a, the outlier of midostaurin for acute myeloid leukemia with an OS benefit of 49.1 months was excluded for visualization purposes. Abbreviations: FDA, US Food and Drug Administration; OS, overall survival; PFS, progression-free survival.

In our recent study, we reviewed and meta-analyzed the clinical evidence and benefit of all new 124 cancer drugs among their 374 indications approved by the US Food and Drug Administration between 2003 and 2021 [2]. The uniqueness of this study lays in the breadth of analyzed indications – we assessed both initial drug approvals and all their supplemental indications. Modern oncologic drugs, such as immune-therapies and targeted agents, are regularly used across multiple cancer diseases; this is the first study that evaluates the benefit of these supplemental indication approvals. Across a total of 234 randomized-controlled trials, we found that new drugs improved OS by a median of 2.80 months and PFS by 3.30 months compared to the control (Figure 1). Compared to the frequently used terminology used to described cancer drug – “breakthrough”, “game-changer”, or “cure” – this median survival benefit appears marginal. However, cancer treatment has become a stepwise process entailing multiple lines of therapy and a combination of different therapeutic regimens. Therefore, the cumulative benefit of all cancer drug indications that have come to the market over the past two decades is substantial and shall not be underestimated. Interestingly, Figure 1 further shows a skewed distribution of the survival benefit – from 2003 to 2021 only 16 drug indications extended OS by more than 6 months [3]. Hence, the increased OS and PFS is driven primarily by a handful of drugs. For example, cetuximab extended survival by 19.7 months for patients with squamous cell carcinoma of head and neck, encorfenib by 16.7 months for patients with melanoma, and olaratumab by 11.8 months for patients with soft tissue sarcoma. Pharmaceutical companies and academic institutions should, therefore, particularly concentrate development efforts on these scientific breakthroughs delivering a meaningful survival benefit for patients besides the continuous incremental improvements offered by new “me-too” cancer drug indications. Thereby the burden cancer poses for society can be further reduced. In summary, cancer drug development is a continuously evolving iterative process with many incremental improvements that nourish on few scientific breakthroughs transforming cancer therapy [3].

Interestingly, we found a seemingly greater benefit for initial drug approvals compared to supplemental indication approvals. However, in this study we show that initial drug approvals are based on small, non-robust clinical trials, e.g. single-arm trials, with inadequate comparators which could overstate a drug’s clinical benefit. We demonstrate that after a drug has been tested in larger randomized controlled trials comparing the new medication in a first-line setting to the current standard of care, the measured clinical benefit decreases. Whilst the development of drugs across different diseases, lines of therapy, and in combination with other agents has become the norm, it poses several challenges for insurers and payers to decide on a drug’s coverage, reimbursement, and pricing across all its multiple clinical indications. In the US a single price is assigned to drugs with multiple indications [4]. In our article, we therefore present and discuss indication-specific coverage, reimbursement, pricing strategies that are currently used in the Europe, Canada, and Australia [4, 5] to better align the value and pricing of multi-indication cancer drugs.

Besides investing in new anti-cancer therapies, which have led to substantial medical advances, we must now also focus on other aspects of cancer as a disease of the elderly [6]. With declining mortality rates and stable/declining incidence rates for most cancer entities, the number of cancer survivors is increasing [6]. Yet, for many cancer entities, there remains a lack of personalized, effective, and equitable follow-up strategies [7]. Research should therefore not only concentrate on the prevention and treatment of cancer, but also on post-treatment strategies to permit the early detection of recurrences, metastasis, and long-term side-effects resulting from anti-cancer therapy.

In our article, we find that most drugs were approved for the “large” tumor entities: lung cancer (15%), leukemia (12%), lymphoma (11%), skin cancer (8%), and breast cancer (8%). Despite the significant biotechnological advances for these large cancer entities over the past two decades, many patients suffering from rare tumors remain without any treatment options. Particularly most ultra-rare cancers continue to be a death sentence for patients. However, the authors are hopeful that the emerging wave of gene and cell therapies combined with novel rare disease policies will be able to stimulate and address the unmet medical needs for therapies against ultra-rare cancers [8, 9].
